# Seasonal activity of ticks infesting domestic dogs in Bejaia province, Northern Algeria

**DOI:** 10.4102/ojvr.v86i1.1755

**Published:** 2019-10-17

**Authors:** Rosa Kebbi, Mohamed Nait-Mouloud, Lila Hassissen, Abdelhanine Ayad

**Affiliations:** 1 Department of Environment Biological Sciences, Faculty of Nature and Life Sciences, University of Bejaia, Bejaia, Algeria; 2 Private Veterinary Practice, Sidi-Ahmed district, Bejaia, Algeria

**Keywords:** prevalence, dynamic, ticks, dogs, Bejaia province

## Abstract

This epidemiological study aimed to determine the species of tick infestation in dogs, their prevalence and dynamic in the Bejaia province, northeastern Algeria. A total of 631 dogs were examined from different localities of the Bejaia province between March 2016 and February 2017. Of the 631 examined dogs, 15% were infested with one or more tick species. A total of 339 adult ticks were collected and identified, including 199 male tick species and 140 female tick species. Our results revealed that most of these were *Rhipicephalus* species, with *Rhipicephalus sanguineus* (51.32%) being the most prevalent followed by *Rhipicephalus bursa* (35.1%) and *Rhipicephalus turanicus* (12.98%). *Ixodes ricinus* represented only 0.6% of all ticks collected. The highest infested seasons were spring (22.55%) and summer (22.54%) and the lowest infested seasons were autumn (8.62%) and winter ( 0.9%). There is no significant difference between the sex of the animal and the prevalence of infestation (*p* = 0.837). Also, the prevalence of infestation by ticks in young animals was higher than that in adult animals (*p* = 0.550). A significant difference between the prevalence of infestation and animal breed was observed (*p* = 0.042). This study is the first epidemiological investigation conducted on the prevalence of hard ticks infesting domestic dogs in Bejaia (northeastern Algeria) based on conventional methods. It is therefore necessary to implement an effective tick control strategy during infestation periods in order to prevent vector-borne diseases.

## Introduction

After mosquitoes, ticks are the second most important obligate haematophagous arthropods that parasitise all classes of vertebrates for their blood meal in almost all regions of the world, particularly in Africa (Laamri et al. 2012; Xhaxhiu et al. 2009). They can also crucially transmit to animals, especially dogs, a large number of protozoa and bacteria, such as *Babesia, Theileria* and *Anaplasma* spp. (Gray et al. 2013; Marquez-Jimenez et al. 2005). The seasonal dynamics of ticks is likely to affect the transmission of pathogens. Several ecological factors can influence the survival and development of ticks, especially temperature, relative humidity and vegetation cover (Sahibi & Rhalem 2007). All tick species are significant disease vectors and the increased incidence of these diseases is mainly attributed to climate change that affects ticks directly or indirectly (Gray et al. 2009). Ticks are highly climate-sensitive arthropods, and all stages of their life cycle depend on a complex combination of climatic variables. The presence of hosts and vegetation greatly modulate the dynamics of their populations. However, vegetation is a major modifier of local climatic conditions, to which ticks must adapt for their development and survival (Estrada-Peña, Ayllón & De La Fuente 2012).

Dogs are the most commonly owned companion pets worldwide (Moriello 2003). They are the most successful canids, adapted to human habituation, which contribute to the physical, social and emotional well-being of their owners (Dohoo et al. 1998). It is possible that dogs carry ticks in the domestic environment and transmit these to humans, which may constitute a major concern for public health (Dantas-Torres, Chomel & Otranto 2012).

In Algeria, very few studies have been conducted on the vectorial role of ticks that infest dogs, except the inventory carried out by Matallah et al. (2013). In terms of biodiversity and specific biology, *Ixodidea* fauna is not sufficiently known in Algeria (Meddour & Meddour 2006). Because of certain geographical specifications and the probable presence of different types of ticks infesting companion animals, epidemiology studies of ticks in dogs are extremely important to plan the approaches of pest management. This study aimed at determining the species of tick infestation in dogs, their prevalence and dynamics by performing an epidemiological study in the Bejaia province (northeastern Algeria).

## Material and methods

### Study area and dogs

**FIGURE 1:**
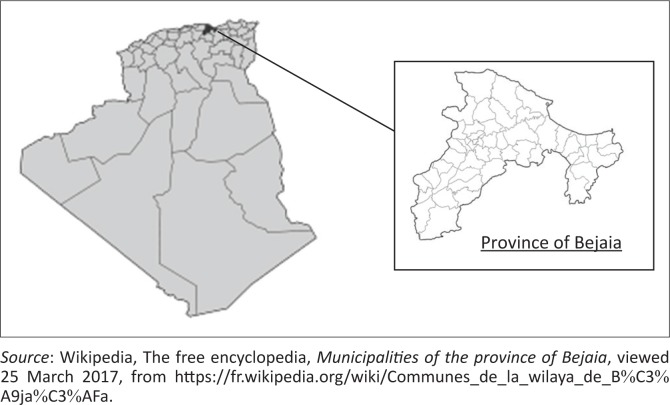
Map of the study area, Bejaia (Northern Algeria, latitude 36°43’N and longitude 5°04’E).

The study was carried out in the Bejaia province of Algeria (36°43’N, 5°04’E) (Figure 1) from March 2016 to February 2017. The province has four distinct seasons: winter (January to March), spring (April to June), summer (July to September) and autumn (October to December). The annual rainfall in the region during the study period ranged from 679 mm to 821 mm. The mean maximum summer temperature was 29.9 °C (August) and the mean minimum winter temperature was 6.4 °C (January) during the study period (Table 1).

**TABLE 1:** Mean ± standard deviation, minimum and maximum value of temperature, rainfall and humidity per month in the Bejaia area, Algeria (2016–2017).

Months	Temperature (°C)		Rainfall (mm)		Humidity (%)
	Mean ± s.d.	Min–Max	Mean ± s.d.	Min–Max	
March 2016	13.1 ± 3.5	8.3–18.3	199.39 ±14.58	0.25–59.94	76.0
April 2016	16.1 ± 1.84	18–21.15	49.27 ± 3.45	0.25–16	80.0
May 2016	18 ± 2.65	13.6–23.3	55.62 ± 6.08	0.25–29.97	76.9
June 2016	22.3 ± 1.8	17.3–26.5	19.06 ± 2.07	1.02–9.91	78.4
July 2016	25.3 ± 1.55	20.2–29.5	-	-	72.9
August 2016	25.2 ± 1.53	20.4–29.9	-	-	73.3
September 2016	23.7 ± 1.8	19–28.8	39.12 ± 4.61	3.05–20.07	74.7
October 2016	22.3 ± 2.58	18.1–28.3	21.59 ± 1.8	0.25–5.08	73.0
November 2016	17 ± 3.13	12.7–22.4	43.19 ± 1.43	1.02–22.1	69.7
December 2016	13.6 ± 1.45	10.1–18.9	41.14 ± 2.5	0.25–9.91	78.8
January 2017	9.9 ± 2.22	6.4–14.7	266.72 ± 16.05	0.5–75.95	74.9
February 2017	13.3 ± 2.25	9.2–18.2	51.81 ± 5.18	0.51–25.91	74.8

*Source:* Climat Bejaia, *Average and total annual climate values*, viewed 25 March 2017, from https://fr.tutiempo.net/climat/2016/ws-604020.html.

s.d., standard deviation.

A total of 631 dogs were selected randomly from different habitats (home and farmhouse) and localities of the Bejaia province. All dogs were presented to a veterinary clinic for different reasons (care, vaccinations, etc.). A dermatological examination was performed by veterinary practitioners and all observations were recorded for each dog examined throughout the study. A complete examination of the skin, visually and by palpation, was done for the presence of ticks. The age of the animals ranged between a few days and over 15 years, with mixed breeds.

### Tick collection and identification

All ticks were removed carefully to ensure that the mouthparts remained intact. The ticks collected were preserved in individually labelled plastic containers containing 70% ethanol. Tick identification was carried out using standard methods at the Laboratory of Animal Biology, University of Bejaia. Each tick was identified using a stereomicroscope (MOTIC, ST-37C-2LOO) according to the standard morphological identification keys (Meddour & Meddour 2006; Walker et al. 2014).

### Statistics analysis

Prevalence was calculated as the ratio between the number of infested dogs and the total number of examined dogs. Abundance was determined as the ratio between the total number of parasitic species and the total number of examined dogs. Infestation intensity was calculated as the ratio between the total number of parasitic species in hosts and the number of infested hosts. Statistical analyses were carried out using the R software version 3.4.4 (http://www.R-project.org/). Tick infestation was analysed using the following factors of variation: sex (male and female), age (young: ≤ 12 months; adult: > 1 year), breed (German Shepherd, Rottweiler, American Staffordshire Terrier, Belgian Shepherd, Dogo Argentino, French Pointer and others) and season (spring, summer, autumn and winter). Statistical analysis was performed using a negative binomial General Linear Model (function ‘glm.nb’ in library ‘MASS’ in R). Tukey’s post-hoc test was performed using the ‘glht’ function from the multcomp package. The values were statistically different when the *p*-value was < 0.05.

### Ethical considerations

Ethical clearance to conduct the study was obtained from the scientific committee of the Faculty of Nature and Life Sciences, University of Bejaia.

## Results

Of the 631 examined dogs, 15% were infested with one or more tick species. A total of 339 mature ticks were collected and identified, including 199 male and 140 female tick species. The overall prevalence of infestation was found to be 15% (94/631) during clinical examination.

In this study, two tick genera, *Rhipicephalus* and *Ixodes*, were identified based on the external morphological characteristics (Table 2). Our results revealed that among the *Rhipicephalus* species, *Rhipicephalus sanguineus* was the most prevalent (51.32%), followed by *R. bursa* (35.1%) and *R. turanicus* (12.98%). *Ixodes ricinus* only represented 0.6% of all ticks collected. The ears and neck are the preferred attachment sites of ticks, with a prevalence of 55.8% and 22.2%, respectively. Mixed infestations with more than one tick species were recorded in the majority of dogs (Table 3).

**TABLE 2:** Number (male and female) and prevalence of various tick species in dogs in the Bejaia province, northeastern Algeria, between March 2016 and February 2017.

Tick species	Number of ticks			Prevalence (%)
	Male	Female	Total	
*Rhipicephalus sanguineus*	93	81	174	51.32
*Rhipicephalus bursa*	74	45	119	35.10
*Rhipicephalus turanicus*	32	12	44	12.98
*Ixodes ricinus*	0	2	2	0.60

**TABLE 3:** Prevalence of infested dogs in the Bejaia province, northeastern Algeria, with mixed tick infestations.

Tick species	Prevalence (%)	Number of infested dogs
*Rhipicephalus sanguineus + Rhipicephalus bursa +Rhipicephalus turanicus*	12.76	12
*Rhipicephalus sanguineus + Rhipicephalus bursa*	13.83	13
*Rhipicephalus sanguineus + Rhipicephalus turanicus*	5.32	5
*Rhipicephalus bursa +Rhipicephalus turanicus*	3.20	3
*Rhipicephalus sanguineus*	30.85	29
*Rhipicephalus bursa*	26.6	25
*Rhipicephalus turanicus*	6.38	6
*Ixodes ricinus*	1.06	1

**FIGURE 2:**
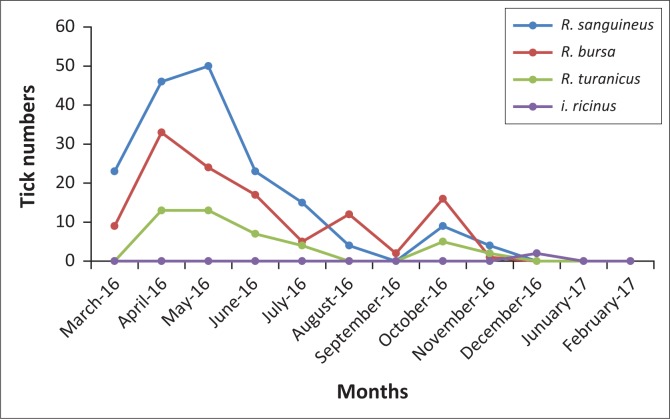
Seasonal abundance of ticks (*Rhipicephalus sanguineus, Rhipicephalus bursa, Rhipicephalus turanicus* and *Ixodes ricinus*) found on owned dogs in the Bejaia province (2016–2017).

Table 4 illustrates the seasonal variation of tick infestation in dogs in the Bejaia province. The total tick prevalence peaked during spring and summer (22.55% and 22.54%, respectively) and the lowest prevalence occurred during winter (0.9%) (*p* < 0.001). The abundance and high intensity of ticks were observed in spring (1.04 and 4.58, respectively) and summer (0.61 and 2.71, respectively), but the lowest abundance and intensity were observed in winter (2.00 and 0.01, respectively) (*p* < 0.05). The monthly variation in the prevalence of infested animals because of *R. sanguineus, R. bursa, R. turanicus* and *I. ricinus* is shown in Figure 2. The prevalence rates recorded of infested dogs by *R. sanguineus, R. bursa* and *R. turanicus* were very high during April and May. As regards *to I. ricinus*, a prevalence rate was observed only in December.

**TABLE 4:** Seasonal variation of tick infestation of dogs in the Bejaia province, northeastern Algeria.

Season	Spring	Summer	Autumn	Winter	*p*
Number of examined dogs	202	142	177	110	-
Number of infested dogs	46	32	15	1	-
Number of collected ticks	211	87	39	2	-
Infestation prevalence†	22.77^a^	22.53^a^	8.47^b^	0.90^b^	< 0.001
Tick abundance‡	1.04^a^	0.61^a^	0.22^b^	0.01^c^	< 0.001
Infestation intensity§	4.58^a^	2.71^a^	2.60^a^	2.00^a^	0.025

† , (Number of infested dogs/total number of examined dogs) ×100.

‡ , Number of collected ticks/number of examined dogs.

§ , Number of collected ticks/number of infested dogs.

a,b,c , Values with different superscripts in the different seasons differ statistically at the same parameter (*p* < 0.05).

The variation of prevalence of infestation by sex, age and breed is shown in Table 5. There was no significant difference between the sex of the animal and the prevalence of infestation (*p* = 0.837) and the age of the animal and the prevalence of infestation (*p* = 0.550). Conversely, the prevalence of infestation by ticks in young animals (≤ 1 year of age) was higher than that in adult animals (> 1 year of age) (*p* = 0.550). Tick prevalence differed significantly between dog breeds (*p* = 0.042).

**TABLE 5:** The variation of the prevalence of tick infestation in dogs with related risk factors (sex, age, breed and season) in the Bejaia province.

Risk factors	Number of examined dogs	Number of positive infested dogs	Tick prevalence (%)	*p*
**Sex**				
Male	266	41	15.79^a^	0.837
Female	365	53	14.29^a^	
**Age**				
Young	443	59	14.00^a^	0.550
Adult	188	35	17.02^a^	
**Breed**				
German Shepherd	210	36	17.14^a^	0.042
Rottweiler	62	16	25.81^a^	
American Staffordshire Terrier	154	12	8.44^a^	
Belgian Shepherd	56	8	14.29^a^	
Dogo Argentino	24	6	20.83^a^	
French Pointer	13	5	38.46^a^	
Others†	112	11	9.82^a^	
**Season**				
Spring	202	46	22.55^a^	< 0.001
Summer	142	32	22.54^a^	
Autumn	177	15	8.62^b^	
Winter	110	1	0.90^b^	

Note: Age (young: ≤ 1 year old; adult: > 1 year old), sex (male and female), breed (German Shepherd, Rottweiler, American Staffordshire Terrier, Belgian Shepherd, Dogo Argentino, French Pointer and others), season (spring, summer, autumn and winter).

† , Others: Blue Gascony Basset, Beagle, Poodle, Griffon, Great Dane and Shar-Pei.

a, b , Values with different superscripts in the same factor differ statistically (*p* < 0.05).

## Discussion

Ectoparasitic infestation is widespread in wild and domestic animals worldwide, several of which are responsible for diseases (Krčmar et al. 2014; Ranju et al. 2012). In Algeria, several surveys have been conducted to study the population of ticks parasitic in cattle (Aouadi et al. 2017; Boucheikhchoukh et al. 2018; Kouidria et al. 2018); however, few structured and published reports are available relating to ticks infesting dogs. The present epidemiological study, extending over 1 year, was conducted to evaluate the spectrum of tick species involved, the levels of infestations and the seasonal dynamic of these ectoparasites.

This study is the first on ticks infesting domestic dogs in the Bejaia province (northeastern Algeria). Our results revealed that the domestic dogs are infested by a variety of tick species, with one or more tick species per infested dog as described previously (Ebrahimzade, Fattahi & Mohammad 2016; Estrada-Peña et al. 2017; Latrofa et al. 2017; Maurelli et al. 2018). The overall prevalence of infestation was slightly low, which could be a result of difficulties in detecting smaller tick life stages (larvae and nymphs) during clinical examination. Studies performed in different countries have shown that the prevalence of infestation by ticks is significantly variable. The prevalence observed in the current study is higher compared to studies from Iran (8.6%) (Ebrahimzade, Fattahi & Mohammad 2016) and Albania (3.5%) (Kumsa & Mekonnen 2011). However, it is substantially lower than that reported by Matallah et al. (2013) in the Souk-Ahras and El-Kala areas, northeastern Algeria (63% and 37 %, respectively). The low rate observed in this study may be explained by the fact that dogs are better maintained by the use of acaricidal treatments. The prevalence rates suggests that these ticks present a real major health problem for domestic dogs and their owners as supported by several studies (Kumsa & Mekonnen 2011; Rinaldi et al. 2007).

Based on the identification keys from morphological characteristics that are approved for African countries, the current results describe four tick species, with a predominance *R. sanguineus.* This finding is in agreement with the previous reports (Benredjem et al. 2014; Bessas et al. 2016; Dahmani et al. 2015; Leulmi et al. 2016). A similar finding of the predominance of this tick species in infested dogs has been reported by Matallah et al. (2013) from northeastern Algeria. In 2011, Mosallanejad, Alborzi and Katvandi (2011) observed the same tick species in companion dogs in the Ahvaz District, southwestern Iran. Studies performed in different countries have shown that the number of tick species is highly variable. Bryson et al. (2000) recorded six species of ticks from dogs belonging to people in resource-poor communities in South Africa. Also, De Mato et al. (2008) identified nine species of ixodid ticks from dogs in Mozambique. In Ethiopia, Kumsa and Mekonnen (2011) identified two species of ticks, namely, *Amblyomma* spp. and *Haemaphysalis leachi*. The variation reported in previous studies might be attributed to different factors, such as geographical locations, climatic conditions and management practices (Krčmar et al. 2014). Additionally, indirect effects of climate change will impact the number of infected ticks by affecting vegetation (Gray et al. 2009). The correlation between positive cases of vector-borne disease and their geographic distribution, as well as potential risk factors (age, sex, breed, type of dog, habitat and prophylactic treatments), was evaluated previously (Mircean et al. 2012). Also, Lindgren, Tälleklin and Polfeldt (2000) concluded that the relatively mild climate of the 1990s in Sweden was probably one of the primary reasons for the observed increase in density and geographic range of *I. ricinus* ticks. In addition, similar preferred attachment sites of ticks in dogs were found in previous studies (Foldvari & Farkas 2005; Krčmar et al. 2014) compared to our data.

*Rhipicephalus sanguineus*, known as the kennel tick, is the most widespread tick in dogs; however, it can also be found in cattle (Walker et al. 2014). Our results are in agreement with reports by several researchers (De Mato et al. 2008; Horak & Matthee 2003; Neves & Horak 2004). Dantas-Torres and Otranto (2017) noted that the life cycle of *R. sanguineus* is adapted to artificial structures such as human habitations and dog kennels. According to Walker et al. (2014), *R. bursa* is one of the common *Rhipicephalus* species in northern Africa, and their preferred hosts are sheep, goats, cattle and horses. However, in this study, the presence of *R. bursa* could be explained by the fact that the dogs were in contact with domestic ruminants, for example, farm dogs. On the other hand, the adult *R. turanicus* infests a variety of hosts such as cats, sheep, goats and wild carnivores, while immature stages of the species never infest dogs (Horak et al. 2000).

Our result revealed two cases of *I. ricinus* ticks collected from companion dogs in the Bejaia area, Algeria. In North Africa, it is known that the ixodid tick species infest livestock, and their adults are present in large numbers only on livestock (Walker et al. 2014). However, *I. ricinus* has been observed in more than 300 host species, including mammals, birds and reptiles (Gern & Humair 2002). It is considered as a potential vector of many disease agents (Farkas 2002). This result could be explained by the fact that dogs would have been infested in the wild. According to Walker et al. (2014), all stages of the development of *I. ricinus* climb in vegetation for transfer to the host. Moreover, the most favourable conditions for the development of *I. ricinus* are in temperatures that are relatively cold and high levels of humidity. Note that the only cases of *I. ricinus* was found in December.

In this survey, the *Rhipicephalus* species collected from companion dogs has shown mainly spring activity, while *I. ricinus* has a winter activity. This could be ascribed to a variety of climatic conditions in this study region. It has been reported that *R. sanguineus* was present in various Maghreb countries of different bioclimatic zones, in which the adult ticks have a seasonal activity from March to November, with a peak activity in May, and it was absent in winter (Bouattour 2002). Moreover, in other studies, the presence of *R. sanguineus* was reported from June to September (Leulmi 2012). In Morocco, Morel (2000) revealed that the seasonal dynamic of *R. bursa* was unimodal, with a peak in activity during the warm season ranging from March to September; this finding is similar to the results of the present study. Likewise, the *R. turanicus* tick species appears in March and disappears in July (Tsatsaris et al. 2016). As shown in Figure 2, *R. turanicus* has been collected between April and November, with a peak infestation in April and May. Concerning the activity period of *I. ricinus*, it agrees with the results reported previously, that is, autumn–winter (Bouattour, Darghouth & Daoued 1999). In contrast, *I. ricinus* developed in bimodal mode in the temperate countries, with intense and low activity peaks during spring and autumn, respectively. In the UK, the maximum abundance of *I. ricinus* has been observed in April and May and their stages show a lesser resurgence in numbers in late summer (Dobson & Randolph 2011).

In the present study, the overall prevalence of dogs infested by different male tick species was higher than female tick species; however, this was not statistically significant (*p* = 0.837). Similarly, Ebrahimzade, Fattahi and Mohammad (2016) reported no statistically significant difference in the tick burden between the sexes living in the same ecological environments. In addition, Rodriguez-Vivas et al. (2003) suggested that both sexes are susceptible to ectoparasite infestation. In contrast, Mosallanejad et al. (2011) found a significant difference between male and female tick species infested by ectoparasites (35.82% vs. 20.33%, respectively).

The proportion of infestation caused by the total number of ticks varied between two age groups of dogs. It was the highest in young and the lowest in adult dogs (*p* = 0.55). Studies have shown that the susceptibility of tick infestation is greater in young animals than in adult animals (Moghaddar, Shorigeh & Gastrodashty 2001; Raut et al. 2006). The influence of age has also been reported by a study in Tunisia, where the frequency of infestation of adult cattle was higher than that of young animals (Bouattour 2001). These results could be because of the immunity system of infected animals which is less developed at an early age (Dillard, Saari & Anttila 2007).

There is a significant difference between the breed of animal and the prevalence of tick infestation (*p* = 0.042). In contrast, no significant effect to bovine (Benchikh-Elfegoun et al. 2007) and dog (Lorusso et al. 2010) breeds on tick infestation was observed in other studies. On the other hand, a previous study reported that dog breeds have a direct influence on the infestation rate (Silveira, Passos & Ribeiro 2009). Statistical analysis revealed that German Shepherd breeds are the most infested compared to other breeds examined in this study. Smith et al. (2011) found that dogs with medium hair length were twice as likely to have ticks than dogs with short hair (*p* < 0.001). Dogs characterised by long hair could provide a conducive environment for tick survival compared to short-haired dogs. It is easy to detect and remove the ticks in the short-haired dogs than those with long hair. Also, short-haired dogs may be more effective in self-grooming and may remove ticks themselves.

## Conclusion

This study is the first epidemiological investigation conducted on the species of ticks, their prevalence and dynamic in infesting domestic dogs in the Bejaia province (northeastern Algeria) based on conventional methods. Our finding showed the presence of four species infesting dogs, with *R. sanguineus* being the most abundant. The low frequency of ticks in the study area raises concerns about the endemic presence of certain diseases transmitted to dogs. It is therefore necessary to implement an effective tick control strategy during infestation periods in order to prevent vector-borne diseases. Also it is recommended that studies regarding the dynamics of hard ticks in other regions should be conducted to complement the knowledge of *Ixodidea* fauna in Algeria.
